# Astrocytic Cebpd Regulates Pentraxin 3 Expression to Promote Fibrotic Scar Formation After Spinal Cord Injury

**DOI:** 10.1007/s12035-023-03207-z

**Published:** 2023-01-12

**Authors:** Shao-Ming Wang, Jung-Yu C Hsu, Chiung-Yuan Ko, Hsiang-En Wu, Yu-Wei Hsiao, Ju-Ming Wang

**Affiliations:** 1 Graduate Institute of Biomedical Sciences, China Medical University, Taichung, 404333 Taiwan; 2 Neuroscience and Brain Disease Center, China Medical University, Taichung, Taiwan; 3 Department of Neurology, China Medical University Hospital, Taichung, Taiwan; 4 Department of Cell Biology and Anatomy, College of Medicine, National Cheng Kung University, Tainan, Taiwan; 5 Ph.D. Program in Medical Neuroscience, College of Medical Science and Technology, Taipei Medical University, Taipei, Taiwan; 6 TMU Research Center of Neuroscience, Taipei Medical University, Taipei, Taiwan; 7 TMU Research Center of Cancer Translational Medicine, Taipei, Taiwan; 8 Cellular Pathobiology Section, Integrative Neuroscience Research Branch, Intramural Research Program, National Institute on Drug Abuse, NIH/DHHS, Suite 3512, 333 Cassell Drive, Baltimore, MD 21224 USA; 9 Department of Biotechnology and Bioindustry Sciences, College of Bioscience and Biotechnology, National Cheng Kung University, Tainan, Taiwan; 10 Graduate Institute of Medical Sciences, College of Medicine, Taipei Medical University, Taipei, Taiwan; 11 International Research Center for Wound Repair and Regeneration, National Cheng Kung University, Tainan, Taiwan; 12 Graduate Institute of Medicine, College of Medicine, Kaohsiung Medical University, Kaohsiung, Taiwan; 13 Institute of Bioinformatics and Biosignal Transduction, College of Bioscience and Biotechnology, National Cheng Kung University, Tainan, 701 Taiwan

**Keywords:** Fibrotic scar, Cebpd, Ptx3, Spinal cord injury, Astrocyte

## Abstract

Astroglial-fibrotic scars resulted from spinal cord injury affect motor and sensory function, leading to paralysis. In particular, the fibrotic scar is a main barrier that disrupts neuronal regeneration after spinal cord injury. However, the association between astrocytes and fibrotic scar formation is not yet understood. We have previously demonstrated that the transcriptional factor Cebpd contributes to astrogliosis, which promotes glial scar formation after spinal cord injury. Herein, we show that fibrotic scar formation was decreased in the epicenter region in *Cebpd*^−/−^ mice after contusive spinal cord injury and astrocytic *Cebpd* promoted fibroblast migration through secretion of Ptx3. Furthermore, the expression of Mmp3 was increased under recombinant protein Ptx3 treatment in fibroblasts by observing microarray data, resulting in fibroblast migration. In addition, regulation of Mmp3 occurs through the NFκB signaling pathway by using an irreversible inhibitor of IκBα phosphorylation in pretreated fibroblasts. Of note, we used the synthetic peptide RI37, which blocks fibroblast migration and decreases fibroblast Mmp3 expression in IL-1β-treated astrocyte conditioned media. Collectively, our data suggest that fibroblast migration can be affected by astrocytic Cebpd through the Ptx3/NFκB/Mmp3 axis pathway and that the RI37 peptide may act as a therapeutic medicine to inhibit fibrotic scar formation after spinal cord injury.

## Introduction

After spinal cord injury, fibrotic scar formation is a major barrier to neurite regeneration and axon growth, resulting in paralysis [1]. Fibrotic scars, which are composed from multicellular formations, such as type A pericytes, stromal fibroblasts, and peripherally derived macrophages, exist at centralized lesions surrounded by glial scars [2, 3]. In the current study, astroglial-fibrotic scars formed by glial and fibrotic scar suggest that astrocytes may be associated with fibrotic scar formation [4].

Fibrotic scar formation primarily appears in the spinal cord hemisection injury model by promoting fibroblast migration, proliferation, and aggregation [5, 6]. Of note, in a contusion model of spinal cord injury, similar to the hemisection model, fibroblasts begin to proliferate, migrate, and aggregate in the lesion core at 3 days post-injury, accumulating extracellular matrix (ECM) deposition to form fibrotic scar in injured lesion [1, 6]. However, the exact molecular mechanisms that lead to fibrotic scar formation in contusive spinal cord injury remain to be fully elucidated.

CCAAT/enhancer-binding protein delta (Cebpd) is an inflammatory transcription factor that regulates the transcription of multiple inflammatory genes. In the central nervous system, Cebpd is mainly expressed in astrocytes, which promote astrocyte activation, anti-apoptosis, and reactive oxygen species production [7–9]. Our previous study has found that astrocytic *Cebpd* induces the formation of glial scars by attracting neighboring astrocyte migration, leading to impaired functional recovery and decreased white matter sparing [10].

Pentraxin-3 (Ptx3) belongs to the long pentraxin family and is expressed in a variety of cell types, especially astrocytes in the central nervous system [11, 12]. It has been shown that astrocytic *Cebpd* activates Ptx3 expression to inhibit macrophage-mediated phagocytosis [9]. Additionally, secreted Ptx3 in the extracellular environment promotes surrounding cell migration [13], inflammation [14], and is correlated with fibrosis [15]. Currently, it has been reported that Ptx3 activates lung fibroblasts to differentiate myofibroblasts through binding to CD44 in which increase ECM deposition and express fibrotic markers (e.g., fibronectin, collagen I, and α-SMA) to form fibrotic scar [16]. However, the molecular mechanism whereby Ptx3 affects fibrotic scar formation after contusive spinal cord injury has yet to be examined.

Here, we found that a lack of *Cebpd* in a mouse model decreased fibronectin aggregation 14 days post-injury. In fibroblast migration assay, conditioned media from IL-1β-treated *Cebpd*^*−/−*^ astrocytes led to decreased migration of fibroblasts. Furthermore, Ptx3 secretion was decreased by IL-1β-treated astrocytic *Cebpd*^−/−^ cells compared to wild-type primary astrocytes. Similarly, meningeal fibroblast migration was also decreased after pretreatment with IL-1β to knock down Ptx3 in primary astrocyte conditioned media. We demonstrated that Mmp3 can be activated by Ptx3 treatment in meningeal fibroblasts through NFκB p65 activation. The Ptx3 peptide RI37 inhibits meningeal fibroblast migration and downregulates Mmp3 transcription in collected IL-1β-pretreated wild-type astrocyte conditioned media. The results suggest that astrocytic *Cebpd* induces Ptx3 secretion and promotes meningeal fibroblast migration through the Ptx3-p65-MMP3 pathway to form fibrotic scars after contusive spinal cord injury.

## Materials and Methods

### Cell Culture

Primary astrocytes were dissociated from wild-type or *Cebpd*^−/−^ newborn mice by mechanical dissociation. The dissociated cells were filtered through a 70-μm nylon strainer (Millipore, Bedford, MA) and seeded in a poly-L-lysine-coated flask containing the medium as previously described [13]. Primary meningeal fibroblasts were dissociated from the brain meninges of wild-type newborn mice by trypsin digestion. The meningeal fibroblasts were seeded in a poly-L-lysine-coated 10-cm culture dish. Both primary astrocytes and meningeal fibroblasts were maintained in Dulbecco’s modified Eagle’s medium (GIBCO) supplemented with penicillin (100 units/mL), streptomycin (100 μg/mL), and 10% fetal bovine serum (FBS) from HyClone Laboratories (Logan, UT, USA).

### Immunofluorescence Analysis

According to a previous publication with modification [10], frozen sections of the spinal cord from Wang et al [10] were mounted onto glass slides and dissolved in OCT in PBS for 10 min. Sections were then blocked with 5% normal goat serum in PBS containing 0.1% Tween 20 for 1 h at room temperature. The sections were incubated with anti-GFAP, anti-fibronectin, and anti-PTX3 in blocking buffer overnight at 4°C. Following three 10-min PBS washes, sections were incubated with Alexa Fluor 405-, 488-, or 555-conjugated secondary antibodies for 1 h. The sections were washed with PBS for 10 min three times and cover-slipped with ProLong Gold antifade reagent with/without 4′,6-diamidino-2-phenylindole for 10 min, as detected by immunofluorescence microscopy.

### Transwell Migration Assay

The meningeal fibroblasts (5 × 10^5^ cells/ml) were suspended in serum-free media, and then 200 μl of the suspended cells was added to the upper compartment of transwell. Serum-DMEM with or without 2.5 μg/ml PTX3 or conditioned medium from IL-1β-treated astrocytes was placed in the lower compartment of transwell. After incubation for 18 h, the cells on the upper surface of the membrane were wiped with a cotton swab. The cells on the lower surface of the membrane were fixed with 4% paraformaldehyde for 10 min and incubated with 0.1% Tween 20 for 10 min. The lower surface of the membrane was stained with ProLong Gold antifade reagent with 4′,6-diamidino-2-phenylindole for 10 min, and the cells were counted under a microscope.

### Western Blot

Experimental cells were harvested and lysed with modified RIPA buffer, including 50 mM Tris-HCl (pH 7.4), 150 mM sodium chloride (NaCl), 1 mM ethylenediaminetetraacetic acid (EDTA), 1% NP40, 0.25% sodium deoxycholate (NaDOC), 1 mM dithiothreitol (DTT), and 10 mM NaF. Lysates were separated by using a sodium dodecyl sulfate (SDS)-containing 10% polyacrylamide gel, transferred to a polyvinylidene difluoride (PVDF) nylon membrane, and probed with specific primary antibodies at 4 °C overnight. The specific primary antibodies were detected by peroxidase-conjugated secondary antibody incubation at room temperature for 1 h. The signals were revealed by an enhanced chemiluminescence (ECL) Western blot system from Pierce (Rockford, IL, USA).

### Quantitative PCR

Briefly, total RNA was collected and extracted using the TRIsure RNA extraction reagent (Invitrogen). cDNA synthesis was performed by using SuperScript III (Invitrogen). Quantitative PCR (qPCR) was conducted using KAPA SYBR FAST qPCR Master Mix (Life Technologies Corporation and Kapa Biosystems Inc.) and was detected by a CFX Connect Real-Time PCR System (BIO-RAD) with the following pairs of specific primers: mouse Ptx3 (forward): 5′- CCTGCTTTTTGCTCTCTGGT-3′ and Ptx3 (reverse): 5′- TCTCCAGCATGATGAACAGC-3′; mouse Mmp-3 (forward): 5′- TGGAACCTGAGACATCACCA-3′ and Mmp-3 (reverse): GATGGAAGAGATGGCCAAAA-3′. ΔΔCt values were used for statistical analysis, while 2^−ΔΔCT^ values were used to plot the graph for Ptx3 and Mmp3 gene expression in different groups.

### Preparation of Conditioned Media

Conditioned media were harvested from primary cultures of wild-type, *Cebpd*^−/−^ or infected with shLacZ or shPtx3 lentivirus astrocytes. Briefly [10], astrocytes were grown in DMEM with 10% FBS overnight and then treated with or without IL-1β for 6 h in serum-free DMEM. After 6 h incubation, astrocytes were washed by PBS twice. The majority of the IL-1β will be removed. Hereafter, the experimental astrocytes were cultured in serum-free DMEM for 12 h. After 12 h incubation, the conditioned media from the cultures were centrifuged, filtered, and stored at −80 °C until further use.

### Statistical Analysis

Prism (version 8.2, GraphPad Software, Inc., San Diego, CA) was used to perform Student’s *t* test, unpaired *t* test, and one-way or two-way ANOVA. The main effects identified by ANOVA were further analyzed by Tukey’s post hoc or Sidak’s multiple comparisons tests. Unless otherwise specified, data were derived from at least three independent experiments from cultured cells or tissue sections for every group. Data are reported as the mean ± SEM. A *p* value of less than 0.05 was considered significant.

## Results

### Lack of Cebpd in Mice with Spinal Cord Injury Results in Decreased Fibrotic Scar Formation

**Fig. 1:**
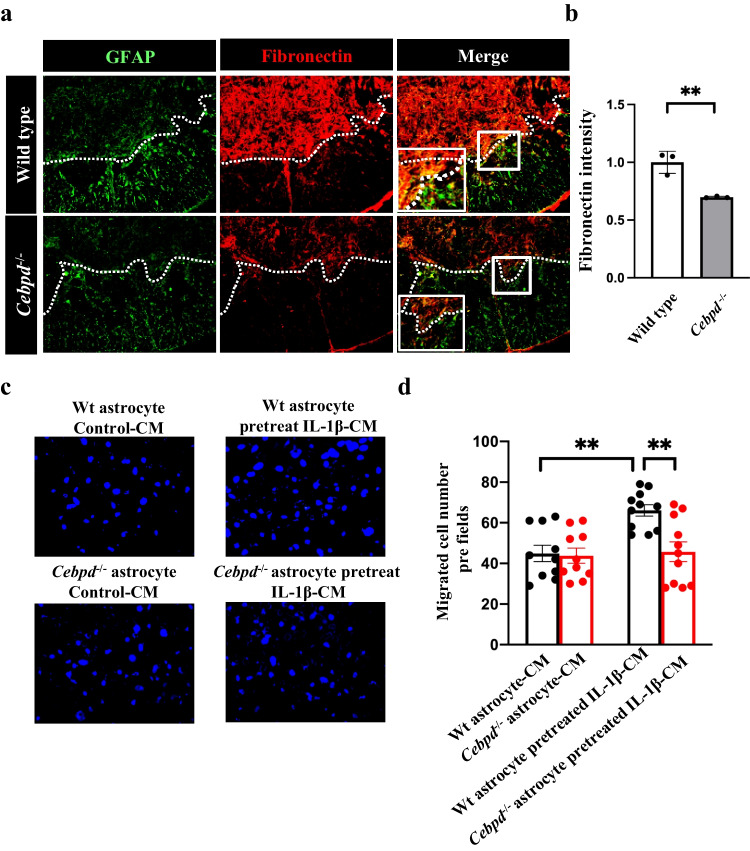
Cebpd deletion in mice decreased fibrotic scar formation after SCI, and Cebpd deletion in astrocytes reduced fibroblast migration in an inflammatory environment. a Fibronectin expression is decreased in *Cebpd*^−/−^ mice. Microscope images showed the expression of Fibronectin (red) and GFAP (green) in the mouse spinal cord after contusive injury. b Summary data from a demonstrate a reduced intensity of Fibronectin. Data are presented as the means ± SEM; *N*=3; two-tailed unpaired Student’s *t* test, *p* = 0.0056, **p < 0.01. c Astrocytic *Cebpd* promotes fibroblast migration. The transwell migration assay was following the “Materials and Methods” section. d Data from c show that astrocytic Cebpd promotes fibroblast migration (bar 1 vs bar 3). In contrast, fibroblast migration was decreased in *Cebpd*-deficient astrocytes pretreated with IL-1β CM (bar 3 vs bar 4); mean ± SEM; *N*=3; two-way ANOVA followed by Sidak’s multiple comparisons test, *p* = 0.0030 and *p* = 0.0036, respectively; **p < 0.01

We have previously shown that Cebpd is mainly expressed in astrocytes, promoting glial scar formation after spinal cord injury [10]. Fibrotic scar formation consists of fibroblasts and is the major barrier to block axon extension during spinal cord injury [1]. Immunofluorescence indicated a reduction in fibronectin expression in *Cebpd*^−/−^ mice compared with wild-type mice after spinal cord injury (Figs. 1a and 1b). The results suggest that astrocytic *Cebpd* contributes to fibrotic scar formation after spinal cord injury. The formation of fibrotic scars is explained below.

### Astrocytic Cebpd Promotes Meningeal Fibroblast Migration

Fibrotic scar formation in contusive spinal cord injury predominantly consists of fibroblasts due to migration to the epicenter from a perivascular cell [1]. Interleukin-1β (IL-1β) is reportedly a major cytokine of inflammation that plays an important role in the consequences of traumatic spinal cord injury [17]. Furthermore, disrupted blood vessels in the epicenter region led to inflammatory cells infiltration and cytokine release [10]. Based on these compelling evidences, IL-1β was used to mimic cellular model of spinal cord injury in this study. We examined whether astrocytic *Cebpd* promotes fibroblast migration through IL-1β treatment. Conditioned medium from IL-1β-pretreated primary astrocytes was used to test fibroblast migration ability. Interestingly, meningeal fibroblasts cultured in conditioned media from IL-1β-pretreated wild-type astrocytes showed increased migration activity. In contrast, the effect was reduced in meningeal fibroblasts treated with conditioned medium from IL-1β-pretreated *Cebpd*^−/−^ astrocytes (Figs. 1c and 1d). The results suggest that astrocytic *Cebpd* might participate in the regulation of IL-1β-induced secretory factors, promoting fibroblast migration.

### Astrocytic Cebpd Regulates Ptx3 Expression and Secretion to Facilitate Fibroblast Migration

**Fig. 2:**
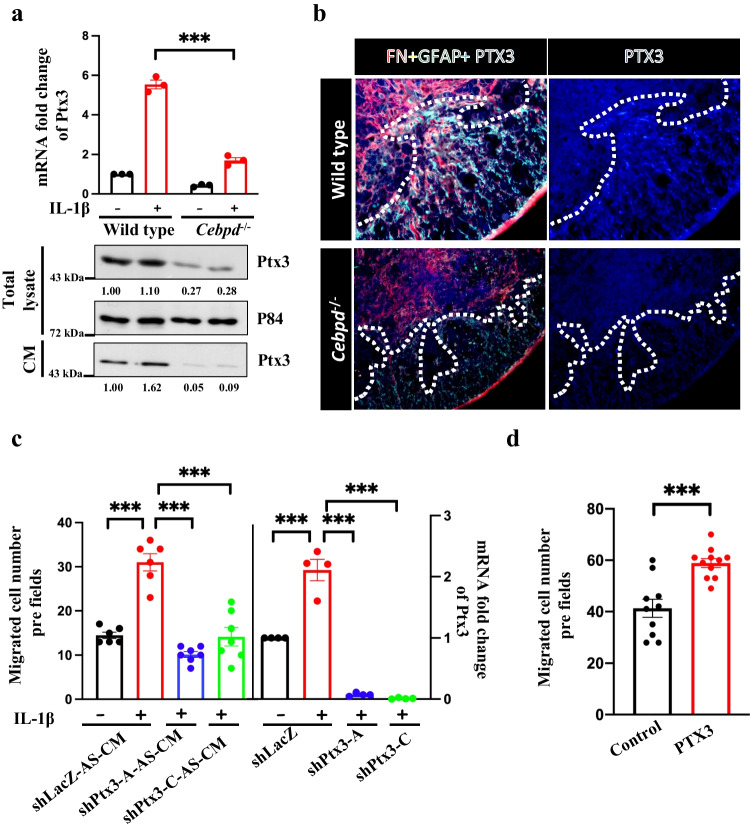
Ptx3 expression and secretion are decreased in Cebpd-deficient astrocytes, which will affect fibroblast migration. a Ptx3 transcription, expression, and secretion were decreased in *Cebpd*^−/−^ astrocytes under IL-1β stimulation. Data are presented as the means ± SEM; *N*=3; Tukey’s multiple comparisons test, *p <*0.0001. b Ptx3 expression (blue) in astrocytes (GFAP, green) was decreased in *Cebpd*^−/−^ mice after spinal cord injury. c Treatment of fibroblasts in CM collected from Ptx3 knockdown primary astrocytes pretreated with IL-1β decreased fibroblast migration compared with conditioned media collected from wild-type astrocytes pretreated with IL-1β. Data are presented as the means ± SEM; *N*=3; two-way ANOVA with Tukey’s multiple comparisons test; *p <*0.0001 (shLacZ-AS-CM vs shPtx3-A-AS-CM; shLacZ-AS-CM vs shPtx3-C-AS-CM). Knockdown of Ptx3 by using shRNA largely reduces Ptx3 transcription (right panel). d Recombinant protein Ptx3-treated fibroblasts increased migration ability. Data are presented as the means ± SEMs; *N*=10; two-tailed unpaired Student’s *t* test, *p*=0.0002, ***p < 0.001

Our previous study showed that Ptx3 can be regulated by Cebpd expression in U373MG cells [9]. In addition, Ptx3 can promote cell migration in breast cancer and head and neck cancer [10, 15]. However, the effect of Ptx3 on meningeal fibroblast migration remains unknown. First, our data showed that Ptx3 transcription and secretion can be activated by IL-1β treatment in primary astrocytes. While Ptx3 was expressed in spinal cord injury sections, IL-1β-induced Ptx3 transcription and secretion were decreased in primary *Cebpd*^−/−^ astrocytes (Fig. 2a). Immunofluorescence indicated that Ptx3 was upregulated and colocalized with GFAP-positive astrocytes in wild-type mice and was attenuated in *Cebpd*^−/−^ mice after spinal cord injury (Fig. 2b).

To examine migration ability, meningeal fibroblasts were treated with conditioned media from IL-1β-pretreated Ptx3 knockdown mouse primary astrocytes. We found that conditional media from Ptx3 knockdown by IL-1β decreased fibroblast migration compared to IL-1β pretreatment control knockdown (Fig. 2c). The Q-RCR data showed that shPtx3 indeed decreased Ptx3 expression (Fig. 2c). On the other hand, fibroblast migration was increased by recombinant protein Ptx3 treatment (Fig. 2d).

### Identification of the Ptx3 Signaling-Regulated Migration Pathway in Meningeal Fibroblasts

**Fig. 3:**
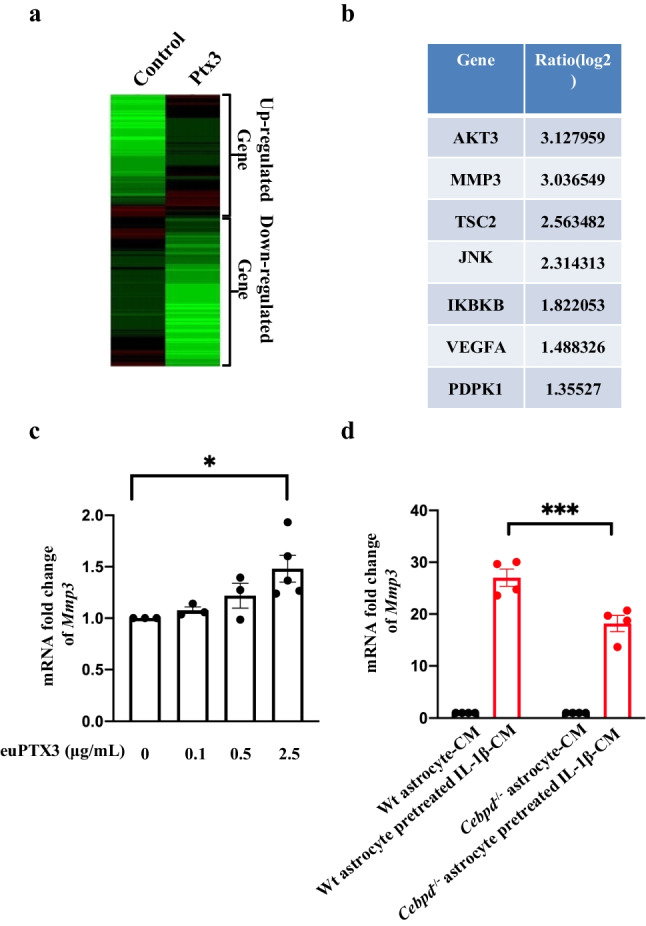
Mmp3 transcription was increased by Ptx3 treatment in fibroblasts. a Total RNA extracted from meningeal fibroblasts treated with or without Ptx3 were examined by microarray. b In Gene Ontology (GO) databases, the upregulated genes of meningeal fibroblasts treated with Ptx3 were shown. c Mmp3 transcription was increased upon euPtx3 treatment. Data from c are presented as the means ± SEM; *N*=3; one-way ANOVA with Dunnett’s multiple comparisons test; *p*=0.0246 (0 vs 2.5 μM euPtx3); *p < 0.05. d Mmp3 mRNA was decreased under conditioned media (CM) treatment obtained from IL-1β-treated *Cebpd*^−/−^ astrocytes compared to CM from IL-1β-treated wild-type astrocytes. Data from d represent the means ± SEM; *N*=3; two-way ANOVA with Tukey’s multiple comparisons test; *p*=0.0007 (WT astrocytes pretreated with IL-1β-CM vs *Cebpd*^−/−^ astrocytes pretreated with IL-1β-CM); ***p < 0.001

The above-mentioned results indicate that Ptx3 can induce meningeal fibroblast migration. How Ptx3-induced genes regulate the cell migration processes in meningeal fibroblasts remains unknown. Transcriptome microarray profiling and comparisons were conducted using meningeal fibroblasts with or without Ptx3 treatment. As shown in Fig. 3a, we found that a total of 935 genes were significantly upregulated and 1356 genes were downregulated by Ptx3 treatment of meningeal fibroblasts. We also used MetaCore analysis to identify potential pathways of the migration-related genes from the GeneGo database. Our data showed that the VEGFA, AKT3, PDPK1, MMP3, IKBKB, TCS2, and JNK were upregulated. Among these genes, MMP3 was significantly increased by Ptx3 treatment (Fig. 3b). We therefore examined whether Mmp3 transcription occurred through Ptx3 stimulation. Our data showed that Mmp3 transcription was upregulated under recombinant protein Ptx3 treatment in meningeal fibroblasts (Fig. 3c). Similarly, meningeal fibroblasts incubated in conditioned media from IL-1β-pretreated wild-type astrocytes showed increased Mmp3 transcription. In contrast, meningeal fibroblasts incubated with conditioned media from IL-1β-pretreated *Cebpd*^−/−^ astrocytes reduced Mmp3 transcription (Fig. 3d). These results demonstrate that Ptx3 promotes fibroblast migration through Mmp3 expression.

### Ptx3 Activates Mmp3 Expression Through the NFκB Pathway

**Fig. 4:**
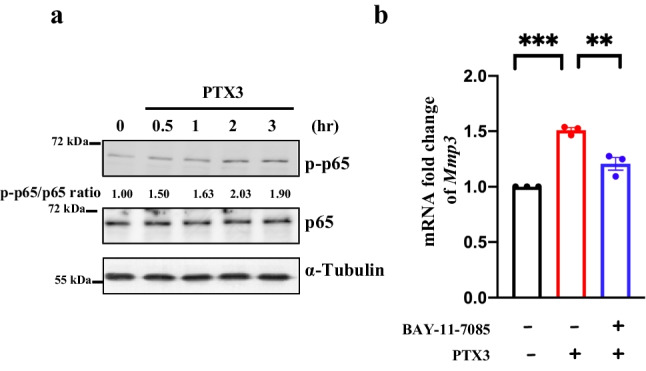
Ptx3 induces Mmp3 transcription via the NFκB pathway. a Ptx3 increases p-p65 activation. P65, phosphorylated p65 (Ser536) and a-Tubulin were analyzed by western blotting. b BAY-11-7085, a IκBα inhibitor, decreases Mmp3 transcription in meningeal fibroblasts under Ptx3 treatment. First, cells were pretreated with BAY-11-7085 for 30 min, then incubated with 2.5 μg/mL euPtx3 for 24 h. Data from b are presented as the means ± SEM; *N*=3; two-way ANOVA with Tukey’s multiple comparisons test; *p*= 0.0001 (control vs euPtx3 groups) and *p*=0.0021 (euPtx3 vs euPtx3+BAY-11-7085 groups); **p <0.01 and ***p < 0.001

To dissect the Ptx3-induced Mmp3 signaling pathway in fibroblasts, a previous study showed that MMP3 expression can be activated by the NFκB pathway [18]. Our results show that Ptx3 activates p65 activation (Fig. 4a) and BAY-11-7085, an inhibitor of NFκB, decreased Mmp3 expression under Ptx3 treatment (Fig. 4b). This result indicates that Ptx3 can increase Mmp3 expression through the NFκB pathway in meningeal fibroblasts.

### The Ptx3-Specific Peptide RI37 Reduces Meningeal Fibroblast Migration and Decreases Mmp3 Expression

**Fig. 5:**
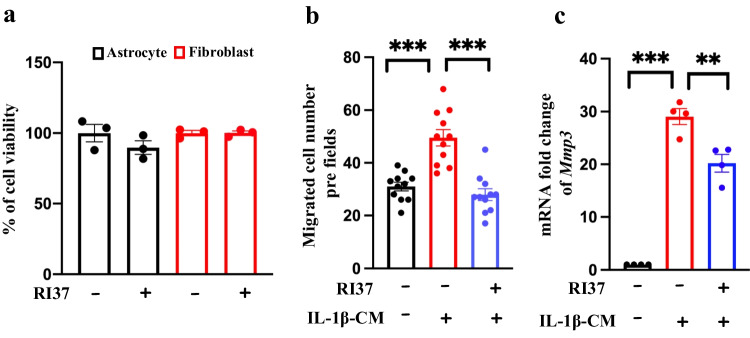
RI37, a synthetic peptide, reduces meningeal fibroblast migration and inhibits Mmp3 transcription under conditioned media harvested from IL-1β-treated wild-type astrocytes. a RI37 did not affect cell viability in primary astrocytes and meningeal fibroblasts. b RI37 reduced meningeal fibroblast migration under conditioned media harvested from IL-1β-treated wild-type astrocytes. Data are presented as the means ± SEM; *N*=3; two-way ANOVA with Tukey’s multiple comparisons test; ****p<0.0001; (control vs IL-1β-CM; IL-1β-CM vs IL-1β-CM+RI37). c RI37 inhibited Mmp3 transcription in fibroblasts growing in conditioned media (CM) from wild-type astrocyte-treated IL-1β. Data are presented as the means ± SEM; *N*=3; two-way ANOVA with Tukey’s multiple comparisons test; ***p<0.001 (control vs IL-1β-CM) and **p=0.0025 (IL-1β-CM vs IL-1β-CM+RI37); **p<0.01

Previous studies have shown that glycosylation of Asn220 plays an important role in Ptx3 regulating cell migration and stemness properties [19]. Thus, our lab synthesized the PTX3 peptide RI37 (amino acids 200-236) and tested their migration ability [19]. To test RI37 cytotoxicity in primary astrocytes and meningeal fibroblasts, our data showed that RI37 did not affect cell survival after RI37 treatment for 24 h (Fig. 5a). We also found that RI37 suppressed meningeal fibroblast migration (Fig. 5b). In the end, we observed that RI37 treatment inhibited Mmp3 expression in meningeal fibroblasts under conditioned media from IL-1β-treated astrocytes (Fig. 5c). The results suggest that Ptx3 acts as a therapeutic target to reduce fibrotic scar formation after spinal cord injury and RI37 can be a therapeutic alternative to treat spinal cord injury patients.

## Discussion

Adequate therapy for spinal cord injury is an unmet medical need due to the resulting functional and emotional impairment and financial burden. Recent studies have shown that fibrotic scars block axon regeneration after contusive spinal cord injury [1, 4]; therefore, understanding the role of fibrotic scar on spinal cord injury is in urgent need. Given the astroglial-fibrotic scar formation mechanism, astrocytes may be associated with fibrotic scar formation which contributes to the interruption of axonal regeneration.

Our previous study showed that Cebpd in astrocytes promotes glial scar formation, which decreases myelination and functional recovery [10]. Here, we found that *Cebpd* deletion in mice decreased fibronectin expression in the epicenter region after spinal cord injury. Furthermore, we found that IL-1β-treated wild-type astrocytes obtained from conditioned media promoted meningeal fibroblast migration; on the other hand, IL-1β-treated *Cebpd*^−/−^ astrocytes obtained from conditioned media reduced meningeal fibroblast migration. We have shown that astrocytic *Cebpd *activates Ptx3 transcription and expression [9]. Herein, we observed that Ptx3 secretion was increased by IL-1β-treated astrocytes. In addition, Ptx3 can induce meningeal fibroblast migration through Mmp3 expression via the NFκB pathway. Finally, the Ptx3-specific peptide RI37 inhibited meningeal fibroblast migration and Mmp3 expression.

Matrix metalloprotease (MMP) activation contributes to migration, inflammation, and scar formation, leading to neurological disability [10, 20, 21]. Previous studies have demonstrated that MMPs, in particular MMP2 and MMP9, promote inflammation and increase cell migration that forms glial scars to block axon regeneration [22, 23]. Additionally, inhibition of MMP8 expression attenuates blood-brain barrier disruption and inflammation after spinal cord injury [24]. In our previous study, we also found that astrocytic Mmp3 recruits surrounding cells to inactivate astrocyte migration, promoting glial scar formation [10]. Notably, Mmp3 can be activated by Ptx3 stimulation in meningeal fibroblasts, which affects fibrotic scar formation. Thus, MMP3 may act as a therapeutic target in contusive spinal cord injury.

The current study demonstrated that Ptx3 expression correlates with cell invasion via the NFκB pathway, regulating AHGEF7 and Rac1 expression in brain tumors [25]. It has been shown that PTX3 downregulation promotes gastric cancer migration and invasion [26]. How these two phenomena apparently have opposite functions is unknown. Our finding here is that Ptx3 promotes meningeal fibroblast migration through p65-regulated Mmp3 expression. These data are consistent with a previous study in which Ptx3 promoted breast cancer stemness, migration, and invasion [19]. More importantly, it is reportedly that the Ptx3 can bind to CD44 transmembrane receptor in lung fibroblasts to activate fibrotic scar-related gene expression, such as fibronectin, collagen I, and α-SMA [16]. Given the relationship between Ptx3 and fibrotic scar formation in spinal cord injury, we speculate that Ptx3 can activate meningeal fibroblasts to express fibrotic scar-related gene expression through CD44 pathway.

Our previous study showed that glycosylation of Asn220 in Ptx3 plays an important role in regulation of inflammation and cell motility [19]. We also examined the effect of RI37, a PTX3 synthesized peptides, on the binding between Ptx3 and the receptor. Synthesized RI37 is included in amino acids 200-236 that cover the glycosylation site, while KT44 (amino acid 255-298), another synthesized peptide of PTX3, is a negative control that does not block cell motility and tumorigenesis [19]. We demonstrated that RI37 can reduce Ptx3 downstream target Mmp3 transcription, and attenuates meningeal fibroblast migration. Collectively, RI37 could be a novel therapeutic reagent to block Ptx3 function, reducing fibroblast migration.

**Fig. 6:**
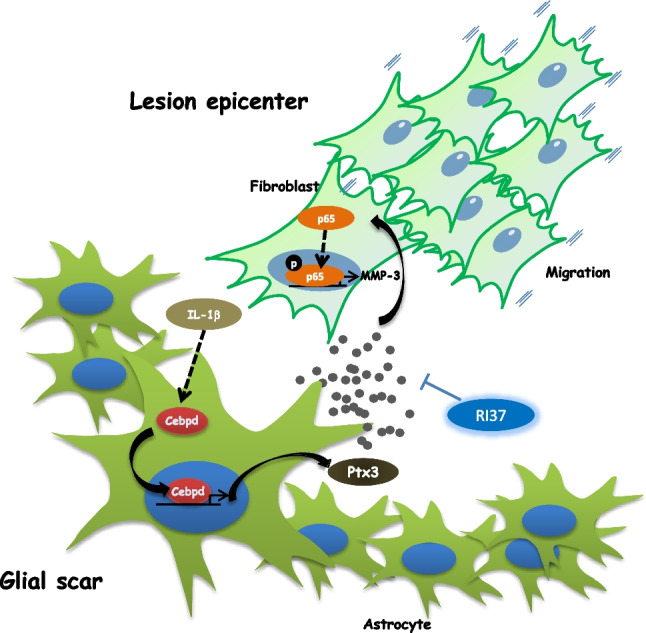
A schematic diagram illustrates the proposed model. Astrocytic Cebpd promotes fibroblast migration to form fibrotic scars through Ptx3 secretion. We hypothesize that Ptx3 induces Mmp3 transcription via the NFκB pathway. RI37, a synthetic peptide, blocks Ptx3-induced fibroblast migration by inhibiting Mmp3 transcription. Consequently, Cebpd promotes fibrotic scar formation through secretion of Ptx3 after spinal cord injury

In conclusion, the transcription factor Cebpd in astrocytes promotes fibrotic scar formation after spinal cord injury due to Ptx3 secretion. The secreted Ptx3 increases Mmp3 transcription through NFκB activation in meningeal fibroblasts, promoting cell migration. The synthesized peptide RI37 blocks Ptx3-induced Mmp3 transcription, which reduces cell migration (Fig. 6). Most importantly, increased Ptx3 secretion may correlate with neurodegenerative disease progression, such as Alzheimer’s disease and Parkinson’s disease [11, 27, 28]. Our results provide the first exploration of a novel mechanistic link between Ptx3 and spinal cord injury and may generate new translational opportunities for spinal cord injury with the synthetic peptide RI37.

## Data Availability

The data that support this study are available from the corresponding authors upon reasonable request.

## Abbreviations

SCI: spinal cord injury
CEBPD: CCAAT/enhancer-binding protein delta
PTX3: Pentraxin-3
MMP: Matrix metalloprotease
EDTA: ethylenediamine tetraacetic acid
NaDOC: sodium deoxycholate
DTT: dithiothreitol
SDS: sodium dodecyl sulfate
PVDF: polyvinylidene difluoride
ECL: enhanced chemiluminescence
CM: conditioned media
